# Growth Forms of *Gardnerella* spp. and *Lactobacillus* spp. on Vaginal Cells

**DOI:** 10.3389/fcimb.2020.00071

**Published:** 2020-02-28

**Authors:** Hyunsul Jung, Marthie M. Ehlers, Remco P. H. Peters, Hennie Lombaard, Mathys J. Redelinghuys, Johanna E. Bezuidenhoudt, Marleen M. Kock

**Affiliations:** ^1^Department of Medical Microbiology, University of Pretoria, Pretoria, South Africa; ^2^Department of Medical Microbiology, Tshwane Academic Division, National Health Laboratory Service (NHLS), Pretoria, South Africa; ^3^Department of Medical Microbiology, Maastricht University, Maastricht, Netherlands; ^4^Wits Obstetrics and Gynaecology Clinical Research Division, Department of Obstetrics and Gynaecology, Rahima Moosa Mother and Child Hospital, University of Witwatersrand/Gauteng Department of Health, Johannesburg, South Africa; ^5^Wits Reproductive Health and HIV Institute, School of Clinical Medicine, University of the Witwatersrand, Johannesburg, Gauteng, South Africa; ^6^School of Health Systems and Public Health, University of Pretoria, Pretoria, South Africa

**Keywords:** bacterial vaginosis, biofilm, *Gardnerella* spp., *Lactobacillus* spp., fluorescence *in situ* hybridization

## Abstract

Bacterial vaginosis (BV) is a common vaginal condition in women of reproductive age. During BV development, BV-associated bacteria may form a polymicrobial biofilm, which predispose women to recurrent BV. The aim of the study was to investigate the growth forms of *Gardnerella* spp. and *Lactobacillus* spp. and to determine the association between the bacterial growth forms and clinical characteristics [urinary tract infection (UTI) symptoms, human immunodeficiency virus (HIV) infection and abnormal vaginal discharge] in women attending a tertiary hospital in Pretoria, South Africa. A first-void urine specimen was collected from 196 women and BV was diagnosed using the Nugent scoring and the Ison-Hay criteria (vaginal smear microscopy). Fluorescence *in situ* hybridisation (FISH) was performed to classify the growth forms [“dispersed” or “biofilm”]. Bacterial cells were categorized as “dispersed” if cells were scattered separately and as “biofilm” if bacterial aggregates on the vaginal epithelial cells were observed. BV was detected in 52 women (52/196; 27%) and in these women, *Gardnerella* spp. were predominantly present in biofilms (46/52; 88% for Nugent scoring; and 45/50; 90% for Ison-Hay criteria), whereas *Lactobacillus* spp. were predominantly present in a dispersed form (38/52; 73% for Nugent scoring; and 37/50; 74% for Ison-Hay criteria). The odds of having BV increased when *Gardnerella* biofilms were present (*p* < 0.001), whereas the opposite was observed for *Lactobacillus* biofilms (*p* = 0.001). Neither *Gardnerella* spp. or *Lactobacillus* spp. (both dispersed or biofilms) had an association with the presence of UTI symptoms, HIV coinfection or abnormal vaginal discharge. In conclusion, this study demonstrated and confirmed that *Gardnerella* biofilms are associated with BV and that *Lactobacillus* spp. may form biofilms to protect against BV.

## Introduction

Bacterial vaginosis (BV) is the most common vaginal disease in women of reproductive age (Kenyon et al., 2013). BV may have a devastating impact on women's reproductive health due to its association with preterm birth (Leitich et al., 2003; Shimaoka et al., 2019), miscarriages (Hay et al., 1994) and tubal infertility (Wilson et al., 2002; van Oostrum et al., 2013). Furthermore, BV has been suggested to increase the risk of acquisition of sexually transmitted infections (STIs) (Allsworth and Peipert, 2011) and human immunodeficiency virus type 1 (HIV-1) (Atashili et al., 2008; van de Wijgert et al., 2008).

BV is a polymicrobial dysbiotic condition, which is characterized by a decrease in the normally resident lactic-acid producing vaginal bacteria (usually *Lactobacillus* spp.) and an overgrowth of vaginal anaerobic bacteria (Hillier et al., 1993; Fredricks et al., 2005; Ravel et al., 2013). Although the exact pathogenesis of BV remains unknown, two main hypotheses are currently in debate to explain the BV etiology—that BV is triggered by the initial establishment of the “key bacteria” such as virulent strains of *Gardnerella* spp., *Prevotella bivia, Atopobium vaginae* and *Megasphaera* type 1; or it is caused by the sexual introduction of the polymicrobial community that predominantly consists of the above-mentioned “key bacteria” (Srinivasan and Fredricks, 2008; Muzny and Schwebke, 2013; Muzny et al., 2018, 2019). *Gardnerella* spp. have long been suggested as an etiological agent of BV since its discovery (described as *Haemophilus vaginalis*) by Gardner and Dukes (1955), but its role in BV etiology has remained controversial over the years because of its presence in the microbiota of “healthy” women (Zhou et al., 2004; Ravel et al., 2011; Shipitsyna et al., 2013). This controversy could partially be explained by the evidence that four different genotypes/clades (or 13 genomic species) may exist within the genus *Gardnerella* (Santiago et al., 2011; Ahmed et al., 2012; Vaneechoutte et al., 2019), and that there are differences in virulence potential between avirulent and virulent strains of *Gardnerella* spp. (Harwich et al., 2010; Janulaitiene et al., 2018).

During the initial establishment of BV, the BV-associated bacteria such as virulent strains of *Gardnerella* spp. may adopt the biofilm mode of growth (Swidsinski et al., 2005, 2014). The formation of a polymicrobial biofilm not only allows BV-associated bacteria to survive and to persist against the antibiotic therapy, but also provides a shelter to tolerate hydrogen peroxide (H_2_O_2_) and lactic acid produced by *Lactobacillus* spp. (Patterson et al., 2007; Swidsinski et al., 2008; Alves et al., 2014). Among many BV-associated bacteria that can form biofilms, virulent strains of *Gardnerella* spp. and *A. vaginae* are the predominant bacterial species actively involved in the formation of a polymicrobial BV biofilm (Swidsinski et al., 2005, 2014; Hardy et al., 2016). *Gardnerella* spp. are hypothesized as the “early colonizer species” that establishes a baseline for the formation of a BV biofilm, because of its strong initial adhesion ability and high biofilm formation capacity (Patterson et al., 2010; Machado et al., 2013a,b; Castro et al., 2015). Anaerobes such as *P. bivia, A. vaginae* and *Megasphaera* type 1 may then co-exist as a “second colonizer species” in a biofilm formed by *Gardnerella* spp. (Swidsinski et al., 2005; Hardy et al., 2016; Muzny et al., 2018, 2019; Castro et al., 2019).

As biofilms allow *Gardnerella* spp. and *A. vaginae* to persist after metronidazole treatment in recurrent BV (Bradshaw et al., 2006; Swidsinski et al., 2008), it could be important to study the growth form of BV-associated bacteria (especially BV biofilms), which is useful in the development of the treatment strategies. One approach to visualize the bacterial growth form and the bacterial composition in biofilms is by using fluorescence *in situ* hybridization (FISH) (Hardy et al., 2016). Many previous studies have successfully used FISH to demonstrate the importance of a polymicrobial biofilm in BV; however, none of these studies have directly compared each growth form of BV-associated bacteria with Ison-Hay criteria and clinical characteristics such as HIV status and urinary tract infection (UTI) symptoms (Swidsinski et al., 2005, 2014; Hardy et al., 2016). Therefore, the present study aimed to investigate the growth forms of BV-associated bacteria (*Gardnerella* spp. and *A. vaginae*) and *Lactobacillus* spp. on the vaginal epithelial cells in first-void urine specimens of women attending a tertiary hospital in Pretoria, South Africa, as well as to determine the association between bacterial growth forms and clinical characteristics (HIV status, presence of UTI symptoms and presence of abnormal vaginal discharge) in BV-positive women.

## Materials and Methods

### Study Setting and Participants

Non-pregnant women attending a gynecology clinic and pregnant women attending a maternal and fetal unit (MAFU) at a tertiary hospital in Pretoria, South Africa were recruited from May 2014 to October 2014. The gynecology clinic provides care for outpatients who have gynecological conditions that cannot be treated at local clinics or have abnormal Pap smear test results, while the MAFU provides specialized care for pregnant women with high risk pregnancies. All adult women (18–75 years-old – see Table 1) were eligible to participate in the study if they gave written informed consent. Women were not enrolled in the study if any antibiotics were consumed at least two weeks prior to sample collection and if they menstruated at the time of sample collection or had abnormal vaginal bleeding. All procedures performed in this study involving human participants were in accordance with the ethical standards of the institutional research ethics committee (Faculty of Health Sciences Research Ethics Committee) and with the 1964 Helsinki declaration and its later amendments or comparable ethical standards. The study was approved by the Faculty of Health Sciences Research Ethics Committee, University of Pretoria (Ethics reference no.: 117/2014). Basic demographic and clinical data were obtained using a questionnaire. The UTI symptoms were defined as any of the following: frequent urination, hematuria, burning sensation, dysuria and nocturia.

**Table 1:** Demographic, clinical and microbiological characteristics of the women included in the study.

**Clinical characteristics**	**Non-pregnant women (*n* = 129)**	**Pregnant women (*n* = 67)**	***p*-value^a^**
**Age**			
Median (range, years)	39 (18–75)	29 (18–42)	**< 0.001**
**Ethnic groups**			
African (black)	114 (88%)	56 (84%)	0.73^b^
Caucasian (white)	12 (9.3%)	7 (10%)	
Indian	3 (2.3%)	2 (3%)	
Inter-racial/multi-racial	0 (0%)	2 (3%)	
**Clinical symptoms**			
Abnormal vaginal discharge			
Present	22 (17%)	12 (18%)	0.88
Absent	107 (83%)	55 (82%)	
UTI symptoms^c^			
Present	28 (22%)	18 (27%)	0.42
Absent	101 (78%)	49 (73%)	
**HIV^d^ status**			
Positive	67 (52%)	10 (15%)	**<0.001^b^**
Negative	57 (44%)	57 (85%)	
Unknown	5 (3.9%)	0 (0%)	
**Vaginal microbiota (Nugent scoring)**			
Nugent score 0–3 (normal vaginal microbiota)	69 (53.5%)	50 (72%)	**0.016**
Nugent score 4–6 (intermediate vaginal microbiota)	20 (15.5%)	5 (7%)	
Nugent score 7–10 (BV^e^)	40 (31%)	12 (20%)	
**Vaginal microbiota (Ison-Hay criteria)**			
Class I (normal vaginal microbiota)	62 (48.1%)	48 (72%)	**0.013^g^**
Class II (intermediate vaginal microbiota)	24 (18.6%)	8 (12%)	
Class III (BV)	39 (30.2%)	11 (16%)	
Class IV (Gram-positive cocci only)	1 (0.8%)	0 (0%)	
Class 0 (Epithelial cells with no bacteria)^f^	3 (2.3%)	0 (0%)	

a *χ^2^ test for independence. Significant p-values (<0.05) are indicated in bold*.

b *For African vs. Caucasian and for HIV-positive vs. HIV-negative*.

c *Urinary tract infection symptoms: frequent urination, hematuria, burning sensation, dysuria, nocturia*.

d *HIV: Human immunodeficiency virus*.

e *BV: bacterial vaginosis*.

f *Equivalent to a Nugent score of 4 (intermediate vaginal microbiota)*.

g *Excluding classes IV and 0*.

### Classification of Vaginal Smears According to Nugent Score and Ison-Hay Criteria

After the informed consent was obtained, a vaginal swab was collected by a physician and a smear was prepared on a frosted microscope slide (Merck KGaA, Darmstadt, Germany) immediately after the swab collection. Upon arrival at the laboratory, the vaginal smears were heat-fixed and Gram stained. Following Gram staining, vaginal smears were read and scored using the Nugent scoring system (Nugent et al., 1991) and Ison-Hay criteria (Ison and Hay, 2002). For the Nugent scoring system, a score of 0–10 is given based on the presence or absence and counted number of three bacterial morphotypes: (i) Gram-positive rods (vaginal lactobacilli); (ii) Gram-negative to Gram-variable short rods (*Gardnerella* spp. */Bacteroides* spp.); and (iii) Gram-negative curved rods (*Mobiluncus* spp.) (Nugent et al., 1991). A score of 0 to 3 is considered to be “normal” microbiota, a score of 4 to 6 is considered to be “intermediate” microbiota and a score of 7 to 10 is considered to be “bacterial vaginosis” (Nugent et al., 1991). The Ison-Hay criteria categorize the vaginal smears into five classes of microbiota, namely: (i) class I (normal microbiota, lactobacilli only); (ii) class II (intermediate microbiota, reduced lactobacilli with mixed bacterial morphotypes); class III (bacterial vaginosis, few or absent lactobacilli with mixed bacterial morphotypes); class IV (epithelial cells with only Gram-positive cocci) and class 0 (epithelial cells with no bacteria) (Ison and Hay, 2002). All slides were re-read at a later stage and were examined by a second examiner for quality control purposes.

### Processing of Urine Specimens

A first-void urine specimen for the FISH examination of the bacterial growth forms on the vaginal epithelial cells was collected from each enrolled woman after informed consent was obtained. A first-void urine specimen was selected for practical purposes because it is easier to collect, and the fixed sample can be stored for long periods of time until the FISH assays are performed. Upon arrival at the laboratory, all collected first-void urine specimens were processed prior to performing the FISH assay as described in Swidsinski et al. (2010) with some modifications. In brief, the cells in 1.5 mL of first-void urine were collected by centrifugation (Spectrafuge™ 24D Digital Micro-centrifuge, Labnet International Inc., Edison, NJ, USA) at 6,000 × *g* for 6 min and were fixed in 1 mL of 4% (v/v) formaldehyde (Merck KGaA, Darmstadt, Germany) in 1x phosphate-buffered saline (PBS) buffer (pH 7.2; Gibco^®^, Thermo Fisher Scientific Inc., Waltham, MA, USA) for 1 h. The pellet was washed with 1x PBS buffer (pH 7.2; Gibco^®^, Thermo Fisher Scientific Inc., Waltham, MA, USA) three times and was resuspended in a 1:1 mixture of 1x PBS and ice-cold 100% ethanol (Merck KGaA, Darmstadt, Germany). The fixed cell suspension was stored at 4°C until further analysis.

### The Probe Specificity Test and the FISH Assay

The probe specificity tests were performed on the following probes: (i) GardV (Swidsinski et al., 2005); (ii) Ato291 (Harmsen et al., 2000); (iii) Lab158 (Harmsen et al., 1999); (iv) Enc38a (Frahm et al., 1998); and (v) Eub338 probes (Amann et al., 1990). GardV, Ato291 and Lab158 probes are oligonucleotide probes that were previously used to visualize *Gardnerella* spp., *A. vaginae* and *Lactobacillus* spp. in a study by Swidsinski et al. (2005) (see Table 2 for information regarding the probes used in the FISH assay). As the Lab158 probe targets both *Lactobacillus* spp. and *Enterococcus* spp. (Harmsen et al., 1999), the Lab158-positive samples with a negative signal to the Enc38a probe specific to *Enterococcus* spp. were considered positive for *Lactobacillus* spp. The Eub338 probe is a broad-range oligonucleotide probe that targets eubacteria (Amann et al., 1990). The specificity of each probe was tested using reference strains (*G. vaginalis* ATCC^®^ 14018™ and 14019™; *A. vaginae* ATCC^®^ BAA-55™; *L. crispatus* ATCC^®^ 33820™). For culturing of reference strains, a single pure colony of *A. vaginae* ATCC^®^ BAA-55™ was inoculated in tryptone soya broth (TSB) (Oxoid, Thermo Fisher Scientific Inc., Waltham, MA, USA), *G. vaginalis* ATCC^®^ 14018™ and 14019™ in brain heart infusion (BHI) broth (Lab M Limited, Heywood, UK) and *L. crispatus* ATCC^®^ 33820™ in de Man, Rogosa and Sharpe (MRS) broth (Oxoid, Thermo Fisher Scientific Inc., Waltham, MA, USA). The TSB inoculated with *A. vaginae* ATCC^®^ BAA-55™ and the MRS broth inoculated with *L. crispatus* ATCC^®^ 33820™ were incubated at 37°C for 48 h in an anaerobic jar (bioMérieux, Marcy l'Etoile, France) using an anaerobic gas generator (GENbox anaer, bioMérieux, Marcy l'Etoile, France). The BHI broth inoculated with either *G. vaginalis* ATCC^®^ 14018™ or *G. vaginalis* ATCC^®^ 14019™ was incubated in a carbon dioxide (CO_2_) incubator (HR212UV, Shanghai Lishen Scientific Equipment Co. Ltd., Shanghai, China) with 5% CO_2_ at 37°C for 48 h. The FISH protocol by Amann (1995) with some modifications was followed for hybridization reactions of the oligonucleotide probes. A volume of 2 mL cultured broth was centrifuged (Spectrafuge™ 24D Digital Micro-centrifuge, Labnet International Inc., Edison, NJ, USA) at 6,000 × *g* for 6 min to obtain a pellet. The supernatant was discarded, and the cell pellet was resuspended in 100 μL of PBS buffer (pH 7.2; Gibco^®^, Thermo Fisher Scientific Inc., Waltham, MA, USA). A volume of 100 μL 4% (v/v) formaldehyde (Merck KGaA, Darmstadt, Germany) in a PBS buffer (pH 7.2; Gibco^®^, Thermo Fisher Scientific Inc., Waltham, MA, USA) was added to fixate the cells and the mixture was incubated at room temperature (25 ± 5°C) for 1 h. After incubation, the mixture was washed twice with 1x PBS buffer (pH 7.2; Gibco^®^, Thermo Fisher Scientific Inc., Waltham, MA, USA) to remove the formaldehyde completely. The washed mixture was vortexed (Vortex Mixer VX-200, Labnet International Inc., Edison, NJ, USA) and diluted 1:10 in a PBS buffer (pH 7.2; Gibco^®^, Thermo Fisher Scientific Inc., Waltham, MA, USA). An aliquot of 10 μL of the dilution was added to each well of the polytetrafluoroethylene (PTFE)-coated microscope slide (Thermo Fisher Scientific Inc., Waltham, MA, USA) and the slide was air-dried for 20 min at 50°C (Techne HB-1D, Bibby Scientific Limited, Stone, UK). A volume of 20 μL of 1% (w/v) (10 mg·mL^−1^) lysozyme solution (Sigma-Aldrich Co., St. Louis, MO, USA) was added to each well of the slide prior to hybridization and the slide was incubated for 30 min at 56°C (Techne HB-1D, Bibby Scientific Limited, Stone, UK). After incubation, 8 μL of hybridization buffer [containing 5 M NaCl solution, 1 M Tris-HCl, 10% sodium dodecyl sulfate (SDS) and sterile water] and 1 μL of working solution of each probe (30 ng·μL^−1^ for Cy3 and Cy5 labeled probes; 50 ng·μL^−1^ for 6-FAM labeled probes) were added to the wells. Hybridization of oligonucleotide probes (GardV, Ato291, Lab158, Enc38a, and Eub338) was performed in a hybridization oven (Techne HB-1D, Bibby Scientific Limited, Stone, UK) at 50°C for 16 h. After hybridization, the slides were rinsed briefly with distilled water and the 4′, 6′-diamidino-2-phenylindole (DAPI) stain (PureBlu™ DAPI Nuclear Staining Dye, Bio-Rad Laboratories Inc., Hercules, CA, USA) was added to counter-stain the DNA of bacteria, fungi and host cells present. The slides were incubated in the dark at room temperature (25 ± 5°C) for 15 min. Finally, a few drops of anti-fading mounting agent (ProLong^®^ Diamond Antifade Mountant, Thermo Fisher Scientific Inc., Waltham, MA, USA) were applied and a cover slip was placed on each slide.

**Table 2:** Oligonucleotide sequences of probes that were used in the FISH assay.

**Probe name**	**Target**	**Nucleotide sequence (5′ to 3′)**	**Fluorochrome label**	**References**
**Assay 1**				
Ato291	*Atopobium* cluster	GGTCGGTCTCTCAACCC	Cy3^a^	Harmsen et al., 2000
Eub338	Eubacteria	GCTGCCTCCCGTAGGAGT	6-FAM^b^	Amann et al., 1990
GardV	Probe derived from Bif662 with 0 mismatches to *G. vaginalis*	CCACCGTTACACCGCGAA	Cy5^b^	Swidsinski et al., 2005
**Assay 2**				
Enc38a	*Enterococcus* spp.	CTCTACCTCCATCATTCT	Cy5	Frahm et al., 1998
Eub338	Eubacteria	GCTGCCTCCCGTAGGAGT	6-FAM	Amann et al., 1990
Lab158	*Lactobacillus* spp. and *Enterococcus* spp.	GGTATTAGCA(C/T)CTGTTTCCA	Cy3	Harmsen et al., 1999

a *Cy3 refers to carbocyanine attached at the 3′ end of oligonucleotides*.

b *Cy5 and 6-FAM are attached at the 5′ end of oligonucleotides*.

Two triplex FISH assays (assay 1: GardV-Cy5, Ato291-Cy3, and Eub338-FAM probes; assay 2: Lab158-Cy3, Enc38a-Cy5, and Eub338-FAM probes) were performed without formamide at 50°C for 16 h according to the standard protocol by Amann (1995) with some modifications. An aliquot of 10 μL of the fixed cell suspension was added to each well of the polytetrafluoroethylene (PTFE)-coated microscope slide (Thermo Fisher Scientific Inc., Waltham, MA, USA) and the slide was air-dried for 20 min at 56°C (Techne HB-1D, Bibby Scientific Limited, Stone, UK). The cell smears were dehydrated for 5 min each in an increasing ethanol series [50, 80, and 96% ethanol (v/v)] (Merck KGaA, Darmstadt, Germany). A volume of 20 μL of lysozyme solution (10 mg·mL^−1^) (Merck KGaA, Darmstadt, Germany) was added to each well of the slide prior to hybridization and the slide was incubated for 30 min at 56°C (Techne HB-1D, Bibby Scientific Limited, Stone, UK) to allow better penetration of the oligonucleotide probes. After incubation, hybridization reaction was performed at 50°C (Techne HB-1D, Bibby Scientific Limited, Stone, UK) for 16 h after adding the hybridization buffer and working solution of each probe to the wells as described above. The microscope slides were rinsed briefly after the incubation in the washing buffer [containing 5 M NaCl solution, 1 M Tris-HCl, 10% sodium dodecyl sulfate (SDS), and sterile water] and were incubated at 50°C (Techne HB-1D, Bibby Scientific Limited, Stone, UK) for 10 min. The slides were rinsed briefly with distilled water and the 4′, 6′-diamidino-2-phenylindole (DAPI) stain (PureBlu™ DAPI Nuclear Staining Dye, Bio-Rad Laboratories Inc., Hercules, CA, USA) was added to counter-stain the DNA of bacteria, fungi and host cells present. The slides were incubated in the dark at room temperature (25 ± 5°C) for 15 min. Finally, a few drops of anti-fading mounting agent (ProLong^®^ Diamond Antifade Mountant, Thermo Fisher Scientific Inc., Waltham, MA, USA) were applied and a cover slip was placed on each slide.

### Fluorescence Microscopic Visualization and Bacterial Growth Phenotype

The Zeiss LSM 510 META Laser Scanning Confocal Microscope (Carl Zeiss Microscopy GmbH, Jena, Germany; objective: Plan-Neofluar 100x/1.3 oil immersion lens) and its accompanying software (Version 3.2 service pack 2, Carl Zeiss Microscopy GmbH, Jena, Germany) were used to detect the fluorescence signal of the hybridized oligonucleotide probes. The fluorescent cells were observed using the Plan-Neofluar objective (100x, oil immersion lens; Carl Zeiss Microscopy GmbH, Jena, Germany). An argon laser (detecting excitation at 458, 477, 488, and 514 nm) was used to detect the EUB338 probe, while a helium-neon laser 1 (detecting excitation at 543 nm) and helium-neon laser 2 (detecting excitation at 633 nm) were used for the Cy3-labeled (Ato291 or Lab158) and Cy5-labeled probes (GardV or Enc38a), respectively. Lastly, a laser diode 405 (detecting excitation at 405 nm) was used to observe the cells stained with the DAPI stain. Ten microscopic fields were inspected per each sample to determine the presence or absence of each growth form. The fluorescence signal for species-specific probes (GardV, Ato291, Lab158, Enc38a) was considered positive if a fluorescence signal for the universal bacterial probe (Eub338) was detected simultaneously at the same position.

**Figure 1:**
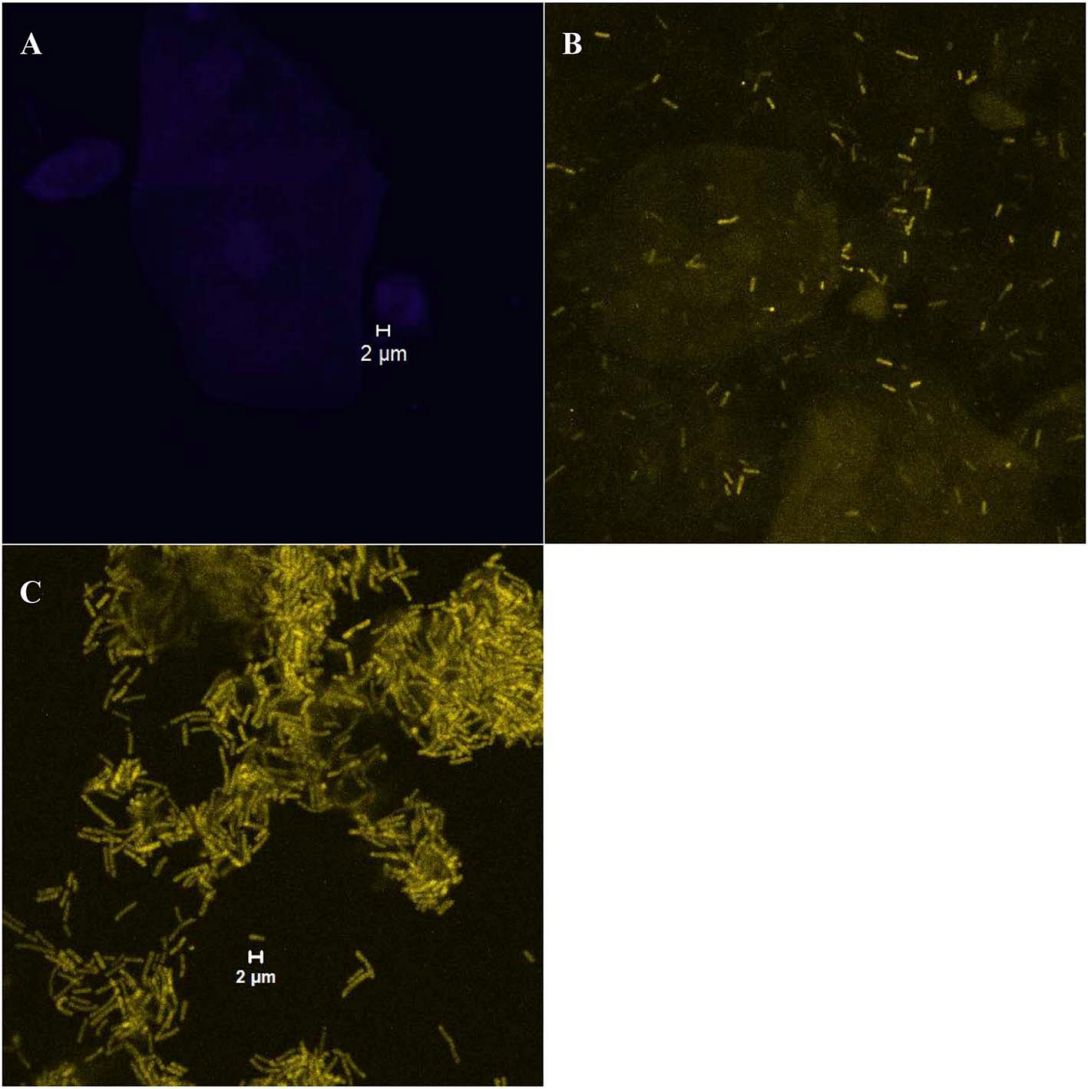
Examples of different bacterial growth forms; **(A)** “absent” category with no bacteria present on vaginal cells (visualized with the laser diode 405 that detects the DAPI stain); **(B)** “dispersed” category [an example of scattered cells of *Lactobacillus* spp. (rods in yellow color) visualized with the helium-neon laser 2 that detects the Lab158-Cy3 probe]; **(C)** “biofilm” category ([an example of the *Lactobacillus* biofilm (rods in yellow color) visualized with the helium-neon laser 2]).

The growth forms of *Gardnerella* spp. and *Lactobacillus* on vaginal cells were classified into two categories, i.e., “dispersed” or “biofilm”. The samples were evaluated as the “dispersed” category if bacterial cells were separately scattered (Figure 1B) and as the “biofilm” category if characteristic bacterial aggregates attached on the vaginal epithelial cells were observed (Figure 1C). The samples with no bacteria present on vaginal cells were classified into the “absent” category (Figure 1A).

### Statistical Analysis

Statistical analysis was performed using the Stata 14.0 software (StataCorp LP, College Station, TX, USA). Descriptive statistics were provided as number (proportion) or median (range); proportions were compared between groups using the χ^2^ test. The agreement between Nugent scoring and the Ison-Hay criteria was measured using the Kappa (κ) value. Forward stepwise logistic regression models based on the likelihood ratio method were fitted with BV status (BV-positive and BV-negative) as outcome, two bacterial growth forms (“dispersed” or “biofilm”) as exposure (dependent variable) and the “absent” category as a reference category. Lastly, the association between two categories of bacterial growth forms and three clinical characteristics (HIV status, presence/absence of UTI symptoms and presence/absence of abnormal vaginal discharges) was determined, in which only BV-positive women were included in the analysis. Fisher's exact test was used for comparing proportions between bacterial growth forms and clinical characteristics as these categories had small sample size. The odds ratios (ORs) were reported with the 95% confidence intervals (CI). All statistical values were considered significant for *p* < 0.05 (95% CI).

## Results

### Study Participants/Population

One hundred and twenty-nine non-pregnant women from the gynecology clinic and 67 pregnant women from the MAFU were included in the study. Women recruited at the gynecology outpatient clinic were generally older (39 years vs. 29 years; *p* < 0.001), more often HIV-infected (52% vs. 15%; *p* < 0.001) and more often diagnosed with BV (31% vs. 20%; *p* = 0.016 for Nugent Score; 30% vs. 16%; *p* = 0.013 for Ison-Hay criteria) (Table 1). The Nugent scoring system revealed that 52 women (27%) were BV-positive, while 25 women (13%) had intermediate vaginal microbiota and 119 women (61%) had normal vaginal microbiota. Nine smears that had normal vaginal microbiota by Nugent scoring (9/119; 7.6%) were assigned to class II by the Ison-Hay criteria; one smear that had intermediate vaginal microbiota (1/21; 5%) were assigned to class III and three smears that were BV-positive by Nugent scoring (3/52; 6%) were assigned to class II. Among the smears that had intermediate vaginal microbiota by Nugent scoring, three smears were assigned to class 0 (3/25; 12%) and one smear was assigned to class IV (1/25; 4%). The κ value of agreement between Nugent scoring and Ison-Hay criteria was 0.88 (95% CI: 0.77 – 0.98).

### Probe Specificity Results

The GardV and Lab158 probes were specific for each target (i.e., had a positive signal for *G. vaginalis* ATCC^®^ 14018™ and 14019™ and *L. crispatus* ATCC^®^ 33820™, respectively and did not cross-hybridize with other reference strains) (Table 3). The Ato291 probe had a positive signal for *A. vaginae* ATCC^®^ BAA-55™; however, the Ato291 probe cross-hybridized with *L. crispatus* ATCC^®^ 33820™. Consequently, the FISH results obtained from the Ato291 probe hybridization were excluded from the analysis.

**Table 3:** Probe specificity test results of the oligonucleotide probes using reference strains.

**Bacterial species**	**Strains**	**Ato291**	**GardV**	**Lab158**	**Enc38a**	**Eub338**
*Atopobium vaginae*	ATCC^®^ BAA-55™	+^a^	-^b^	-	-	+
*Gardnerella vaginalis*	ATCC^®^ 14018™	-	+	-	-	+
*Gardnerella vaginalis*	ATCC^®^ 14019™	-	+	-	-	+
*Lactobacillus crispatus*	ATCC^®^ 33820™	+	-	**+**	-	+

a *(+): presence of hybridization*.

b *(–): absence of hybridization*.

### Growth Forms of *Gardnerella* spp. and *Lactobacillus* spp. on Vaginal Epithelial Cells

*Gardnerella* spp. were mostly present in biofilms (76/196; 39%), whereas *Lactobacillus* spp. were mostly present in a dispersed form (115/196; 59%). Among the samples that contained *Gardnerella* spp. only (i.e., samples without *Lactobacillus* spp.; 16/196; 8.2%), 15 had biofilms (15/16; 94%) and one had a dispersed form (1/16; 6%). Among the samples that contained *Lactobacillus* spp. only (i.e., samples without *Gardnerella* spp.; 98/196; 50%), 60 had a dispersed form (60/98; 61%) and 38 formed biofilms (38/98; 39%). Dispersed *Gardnerella* spp. (9/196; 4.6%) were observed with equal amounts of dispersed form (4/9; 44%) and biofilm (4/9; 44%) of *Lactobacillus* spp., respectively. *Gardnerella* biofilms were mostly observed when *Lactobacillus* spp. were dispersed (51/76; 67%) but some samples with *Gardnerella* biofilms were also observed with *Lactobacillus* biofilms (10/76; 13%). Thirteen samples (13/196; 6.6%) did not contain either *Gardnerella* spp. or *Lactobacillus* spp.

*Gardnerella* biofilms were detected in 88% (46/52) of samples from BV-positive women and no dispersed *Gardnerella* spp. was observed in samples from BV-positive women (Table 4). There was a significant difference (*p* < 0.001) between growth forms of *Gardnerella* spp. and between Nugent score categories. The majority of dispersed *Lactobacillus* spp. (63/115; 55%) and the *Lactobacillus* biofilms (43/52; 83%) were observed in samples from women with normal vaginal microbiota (Table 4). There was also a significant difference (*p* < 0.001) between growth forms of *Lactobacillus* spp. and between Nugent score categories. Similar observations were obtained for the Ison-Hay criteria (Table 4).

**Table 4:** Association of growth forms of *Gardnerella* spp. and *Lactobacillus* spp. in relation to BV status.

**Bacterial growth form**	**Nugent scoring**				**OR^a^ (95% CI)**	***p*-value^d^**	**Ison-Hay criteria^b^**				**OR^c^ (95% CI)**	***p*-value^d^**
	**Total *N***	**Nugent 0–3 (%) (*n* = 119)**	**Nugent 4–6 (%) (*n* = 25)**	**Nugent 7–10 (%) (*n* = 52)**			**Total *N***	**Class I (%) (*n* = 110)**	**Class II (%) (*n* = 32)**	**Class III (%) (*n* = 50)**		
***Gardnerella*** **spp**.												
Absent	111	95 (86^e^)	10 (9)	6 (5)	Ref^f^	**<0.001^g^**	108	91 (84^h^)	12 (11)	5 (5)	Ref^f^	**<0.001^g^**
Dispersed forms	9	8 (89)	1 (11)	0 (0)	-^i^	-	9	8 (89)	1 (11)	0 (0)	-	-
Biofilms	76	16 (21)	14 (18)	46 (61)	27 (11 – 69)	**<0.001**	75	11 (15)	19 (25)	45 (60)	31 (11 – 85)	**<0.001**
***Lactobacillus*** **spp**.												
Absent	29	13 (45^e^)	6 (21)	10 (35)	Ref^f^	**0.002^g^**	27	11 (41^h^)	7 (26)	9 (33)	Ref^f^	**0.003^g^**
Dispersed forms	115	63 (55)	14 (12)	38 (33)	-	-	113	59 (52)	17 (15)	37 (33)	-	-
Biofilms	52	43 (83)	5 (10)	4 (8)	0.17 (0.057 – 0.49)	**0.001**	52	40 (77)	8 (15)	4 (8)	0.17 (0.058 – 0.50)	**0.001**

a *Odds ratio (95% confidence intervals); Exposure: bacterial growth forms; Outcome: BV-positive (Nugent score 7-10) vs. BV-negative (Nugent score 1-3 and 4-6); Entering significance level of 0.05; Removing significance level of 0.1; Only significant OR was displayed*.

b *Class IV and 0 omitted for comparison with Nugent scoring*.

c *Odds ratio (95% confidence intervals); Class IV and 0 omitted for comparison with Nugent scoring; Exposure: bacterial growth forms; Outcome: BV-positive (Class III) vs. BV-negative (Classes I and II); Entering significance level of 0.05; Removing significance level of 0.1; Only significant OR was displayed*.

d *Significant p-values (<0.05) are indicated in bold*.

e *Row percentage between Nugent score categories*.

f *Used as a reference category for OR*.

g *χ^2^ test*.

h *Row percentage between classes I, II, and III*.

i *Not available or not applicable*.

### Association of Biofilms With Bacterial Vaginosis and Clinical Characteristics

The odds of having BV (Nugent score 7–10 or class III) increased when *Gardnerella* biofilms were present (OR 27; CI 11 – 69; *p* < 0.001 for Nugent scoring; and OR 31; CI 11– 85; *p* < 0.001 for Ison-Hay criteria) (Table 4). In contrast, the odds of having BV decreased when *Lactobacillus* spp. were present in biofilms (OR 0.17; CI 0.057 – 0.49; *p* = 0.001 for Nugent scoring; and OR 0.17; CI 0.058 – 0.50; *p* = 0.001 for Ison-Hay criteria). There was no significant relationship between the tested clinical characteristics and the bacterial growth forms (Tables 5, 6).

**Table 5:** Association of growth forms of *Gardnerella* spp. and *Lactobacillus* species in relation to clinical characteristics in BV-positive women (Nugent scoring).

**Bacterial growth form**	**HIV status**				**UTI symptoms**				**Abnormal vaginal discharge**			
	**HIV-negative (%) (*n* = 19)**	**HIV-positive (%) (*n* = 33)**	**OR^a^ (95% CI)**	***p*-value**	**Absent (%) (*n* = 39)**	**Present (%) (*n* = 13)**	**OR^b^ (95% CI)**	***p*-value**	**Absent (%) (*n* = 41)**	**Present (%) (*n* = 11)**	**OR^c^ (95% CI)**	***p*-value**
***Gardnerella*** **spp**.												
Absent	2 (11^d^)	4 (12)	Ref^e^	1.0^f^	4 (10^d^)	2 (15)	Ref^e^	0.63^f^	6 (15^d^)	0 (0)	Ref^e^	0.32^f^
Dispersed	0 (0)	0 (0)	-^g^	-	0 (0)	0 (0)	-	-	0 (0)	0 (0)	-	-
Biofilm	17 (89)	29 (88)	-	-	35 (90)	11 (85)	-	-	35 (76)	11 (100)	-	-
***Lactobacillus*** **species**												
Absent	1 (5^d^)	9 (27)	Ref^e^	0.058^f^	8 (21^d^)	2 (15)	Ref^e^	0.08^f^	7 (17^d^)	3 (27)	Ref^e^	0.53^f^
Dispersed	15 (79)	23 (70)	-	-	30 (77)	8 (62)	-	-	30 (73)	8 (73)	-	-
Biofilm	3 (16)	1 (3)	-	-	1 (3)	3 (23)	-	-	4 (9.8)	0 (0)	-	-

a *Odds ratio (95% confidence interval) adjusted by pregnancy status; Exposure: bacterial growth forms; Outcome: HIV-negative vs. HIV-positive; Entering significance level of 0.05; Removing significance level of 0.1; Only significant OR was displayed*.

b *Odds ratio (95% confidence interval) adjusted by pregnancy status; Exposure: bacterial growth forms; Outcome: UTI symptoms absent vs. UTI symptoms present; Entering significance level of 0.05; Removing significance level of 0.1; Only significant OR was displayed*.

c *Odds ratio (95% confidence interval) adjusted by pregnancy status; Exposure: bacterial growth forms; Outcome: abnormal vaginal discharge absent vs. abnormal vaginal discharge present; Entering significance level of 0.05; Removing significance level of 0.1; Only significant OR was displayed*.

d *Column percentage between bacterial growth forms*.

e *Used as a reference category for OR*.

f *Fisher's exact test*.

g *Not available or not applicable*.

**Table 6:** Association of growth forms of *Gardnerella* spp. and *Lactobacillus* species in relation to clinical characteristics in BV-positive women (Ison-Hay criteria^a^).

**Bacterial growth form**	**HIV status**				**UTI symptoms**				**Abnormal discharge**			
	**HIV-negative (%) (*n* = 19)**	**HIV-positive (%) (*n* = 31)**	**OR^b^ (95% CI)**	***p*-value**	**Absent (%) (*n* = 37)**	**Present (%) (*n* = 13)**	**OR^c^ (95% CI)**	***p*-value**	**Absent (%) (*n* = 40)**	**Present (%) (*n* = 10)**	**OR^d^ (95% CI)**	***p*-value**
***Gardnerella*** **spp**.												
Absent	2 (11^e^)	3 (10)	Ref^f^	1.0^g^	3 (8^e^)	2 (15)	Ref^f^	0.60^g^	5 (13^e^)	0 (0)	Ref^f^	0.57^g^
Dispersed	0 (0)	0 (0)	-^h^	-	0 (0)	0 (0)	-	-	0 (0)	0 (0)	-	-
Biofilm	17 (89)	28 (90)	-	-	34 (92)	11 (85)	-	-	35 (88)	10 (100)	-	-
***Lactobacillus*** **species**												
Absent	1 (5^e^)	8 (26)	Ref^f^	0.065^g^	7 (19^e^)	2 (15)	Ref^f^	0.11^g^	7 (18^e^)	2 (20)	Ref^f^	0.86^g^
Dispersed	15 (79)	22 (71)	-	-	29 (78)	8 (62)	-	-	29 (73)	8 (80)	-	-
Biofilm	3 (16)	1 (3)	-	-	1 (3)	3 (23)	-	-	4 (10)	0 (0)	-	-

a *Class IV and 0 omitted for comparison with Nugent scoring*.

b *Odds ratio (95% confidence interval) adjusted by pregnancy status; Exposure: bacterial growth forms; outcome: HIV-negative vs. HIV-positive; Entering significance level of 0.05; Removing significance level of 0.1; only significant OR was displayed*.

c *Odds ratio (95% confidence interval) adjusted by pregnancy status; Exposure: bacterial growth forms; outcome: UTI symptoms absent vs. UTI symptoms present; Entering significance level of 0.05; Removing significance level of 0.1; only significant OR was displayed*.

d *Odds ratio (95% confidence interval) adjusted by pregnancy status; Exposure: bacterial growth forms; outcome: abnormal vaginal discharge absent vs. abnormal vaginal discharge present; Entering significance level of 0.05; Removing significance level of 0.1; only significant OR was displayed*.

e *Column percentage between bacterial growth forms*.

f *Used as reference category for OR*.

g *Fisher's exact test*.

h *Not available or not applicable*.

## Discussion

The present study set out to investigate the growth forms of *Gardnerella* spp. and *Lactobacillus* spp. on vaginal cells in first-void urine specimens of enrolled South African women with normal vaginal microbiota, intermediate vaginal microbiota and BV using FISH. The findings in this study demonstrate that *Gardnerella* spp. are predominantly present in biofilms on vaginal cells of BV-positive women, while *Lactobacillus* spp. are present in a dispersed growth form. The study also confirms that biofilms formed by *Gardnerella* spp. increase the odds of having BV and that *Lactobacillus* biofilms have the opposite effect.

The presence of *Gardnerella* biofilms in the majority of BV-positive women and the association of *Gardnerella* biofilms with BV observed in this study is a common observation confirmed by previous studies (Swidsinski et al., 2005, 2010; Hardy et al., 2015). It can be hypothesized that *Gardnerella* spp. may prefer to choose the biofilm form of growth during BV as it provides protection against antimicrobial substances like H_2_O_2_ and lactic acid (Patterson et al., 2007), as well as antibiotics/antiseptics (Swidsinski et al., 2008, 2015). Indeed, virulent strains of *Gardnerella* spp. are known strong biofilm formers with good adherence ability, which outcompete other BV-associated anaerobes (Patterson et al., 2010; Machado et al., 2013a; Alves et al., 2014; Castro et al., 2015). For this reason, *Gardnerella* spp. have recently been suggested as the main “early colonizer species” that displace lactobacilli and form a biofilm to create a favorable environment for other BV-associated bacteria such as *Prevotella bivia, A. vaginae* and *Megasphaera* type 1 (Machado et al., 2013a; Swidsinski et al., 2014; Hardy et al., 2015; Muzny et al., 2019).

Interestingly, the present study revealed that *Lactobacillus* biofilms can decrease the odds of having BV in women. The *Lactobacillus* biofilms were observed more frequently in women with normal vaginal microbiota than in BV-positive women (43/52; 83% vs. 4/52; 8% for Nugent scoring and 40/52; 77% vs. 4/52; 8% for Ison-Hay criteria) and had an inverse association with BV (*p* = 0.001 for Nugent scoring; and *p* = 0.001 for Ison-Hay criteria) in this study. These observations could possibly support previous findings that some vaginal lactobacilli may form a biofilm to disrupt and prevent the colonization of pathogenic bacteria (Saunders et al., 2007; Leccese Terraf et al., 2014; Ventolini, 2015). It could also be proposed that lactobacilli may form biofilms to prevent the formation of a polymicrobial BV biofilm. However, lactobacilli were absent in some women with normal vaginal microbiota (13/119; 10.9% for Nugent scoring and 11/110; 10% for Ison-Hay criteria), as previously reported (Zhou et al., 2004; Ravel et al., 2011). According to Ravel et al. (2011) and Zhou et al. (2004), some women may still maintain a “normal” vaginal environment without lactobacilli and that these women may contain BV-associated vaginal bacteria like *A. vaginae, Leptotrichia* spp. and *Megasphaera* spp., which are capable of producing lactic acid.

Lastly, the present study has attempted to determine the association between different bacterial growth forms and three clinical characteristics (HIV status, UTI symptoms and abnormal vaginal discharge) in BV-positive women. However, there was no significant association observed between the bacterial growth forms and tested clinical characteristics. This could be due to a small sample size of BV-positive women.

The present study acknowledges that there are limitations. The first limitation was that the FISH results of *A. vaginae* could not be included in the analysis due to the suboptimal specificity of the Ato291 probe. Cross-hybridization of the Ato291 probe was also observed in a study by Hardy et al. (2015), indicating that use of the peptide nucleic acid (PNA) probe instead of oligonucleotide probe could be useful in future. Another limitation is the usage of the urine specimens for detecting growth forms on the vaginal cells, which might not have adequate amounts of vaginal epithelial cells for the FISH assay as compared to vaginal swabs. Therefore, future studies should utilize the vaginal swab eluates for the FISH assays to improve results. Furthermore, as this study did not determine the clades/subgroups of *Gardnerella* spp., it could be beneficial to perform the clade-specific PCR assays along with the FISH assays to determine associations between the growth forms with different strains of *Gardnerella* spp.

In conclusion, this study demonstrated and confirmed that *Gardnerella* spp. form biofilms on vaginal epithelial cells of BV-positive women, whereas *Lactobacillus* spp. display a dispersed growth form on vaginal cells. This study also revealed that *Lactobacillus* spp. may form biofilms to have a protective role against BV. This study encourages further studies covering a wide array of BV-associated bacteria including *A. vaginae* to uncover the mystery of biofilm formation mechanisms in BV.

## Data Availability Statement

The datasets generated for this study are available on request to the corresponding author.

## Ethics Statement

The studies involving human participants were reviewed and approved by Faculty of Health Science Research Ethics Committee, University of Pretoria (Ethics ref. no. 117/2014). The patients/participants provided their written informed consent to participate in this study.

## Author Contributions

HJ was involved in the concept design, experimental work, data and statistical analysis as well as the writing and editing of the manuscript. ME, HL, MR, and MK were involved in the concept design. ME, RP, HL, MR, and MK critically reviewed the manuscript. RP and JB were involved in the statistical analysis of the acquired data.

### Conflict of Interest

The authors declare that the research was conducted in the absence of any commercial or financial relationships that could be construed as a potential conflict of interest.
